# Supplementary material for: Prevalence and determinants of erectile dysfunction among type 2 diabetes mellitus patients at selected government hospitals in gurage zone: A cross-sectional study
Source: PLoS One. 2025 Apr 21;20(4):e0318908. doi: 10.1371/journal.pone.0318908 (PMC12011261; doi:10.1371/journal.pone.0318908)
Supplement: S1 Table — (DOCX) [file pone.0318908.s001.docx]

S Table 1. Association between variables among T2DM

| variables | category | | No erectile dysfunction frequency(%) | | Erectile dysfunction frequency(%) | | P value |
| --- | --- | --- | --- | --- | --- | --- | --- |
| Age group | < 40 years | | 32(66.7%) | | 41(26.3%) | | P<0.001 |
|  | 40-50 years | | 14(29.2%) | | 58(37.2%) | |  |
|  | >50 years | | 2(4.2%) | | 57(36.5%) | |  |
| Marital Status | Single | | 15(31.2%) | | 26(16.7%) | | P=0.155 |
|  | Married | | 30(62.5%) | | 120(76.9%) | |  |
|  | Separated | | 3(6.2%) | | 9(5.8%) | |  |
|  | Divorced | | 0 | | 1(0.6%) | |  |
| Educational Status | No Formal Education | | 10(20.8%) | | 38(24.4%) | | P=0.464 |
|  | Primary | | 9(18.8%) | | 38(24.4%) | |  |
|  | Secondary | | 13(27%) | | 24(15.4%) | |  |
|  | Diploma | | 8(16.7%) | | 26(16.7%) | |  |
|  | Degree and Above | | 8(16.7%) | | 30(19.2%) | |  |
| Occupational Status | Farmer | | 10(20.8%) | | 40(25.6%) | | P=0.249 |
|  | Merchant | | 12(25.0%) | | 45(28.8%) | |  |
|  | Government | | 12(25.0%) | | 46(29.5%) | |  |
|  | House wife | | 14(29.2%) | | 25(16.0%) | |  |
| Monthly Income (Birr) | <1000 | | 12(25.0%) | | 23(14.7%) | | P=0.135 |
|  | 1000-2000 | | 16(33.3%) | | 45(28.8%) | |  |
|  | >2000 | | 20(41.7%) | | 88956.4%) | |  |
| Physical activity | Yes | | 18(37.5%) | | 65(41.7%) | | P=0.607 |
|  | No | | 30(62.5%) | | 91(58.3%) | |  |
| Alcohol drink | Yes | | 10(20.8%) | | 79(50.6%) | | P<0.001 |
|  | No | | 38(79.2%) | | 77(49.4%) | |  |
| Smoking | Yes | | 11(22.9%) | | 56(35.9%) | | P=0.094 |
|  | No | | 37(77.1%) | | 100(64.1%) | |  |
| Khat Chewing | Yes | | 15(31.2%) | | 93(59.6%) | | P<0.001 |
|  | No | | 33(68.8%) | | 63(40.4%) | |  |
| BMI | Normal | 25(52.1%) | | 24(15.4%) | | P<0.001 | |
|  | Over weight | 10(20.8%) | | 74(47.4%) | |  |  |
|  | obese | 13(27.1%) | | 58(37.2%) | |  |  |
| Duration of diabetes | <5yrs | 29(60.4%) | | 50(32.1%) | | P=0.002 | |
|  | 5-10yrs | 14(29.2%) | | 74(47.4%) | |  |  |
|  | >10yrs | 5(10.4%) | | 32(20.5%) | |  |  |
| Diabetes complication | Yes | 12(25.0%) | | 46(29.5%) | | P=0.547 | |
|  | No | 36(75.0%) | | 110(70.5%) | |  |  |
| Co-existing hypertension | Yes | 23(47.9%) | | 69(44.2%) | | P=0.654 | |
|  | No | 25(52.1%) | | 87(55.8%) | |  |  |
| On hypertensive drug | Yes | 15(31.2%) | | 52(33.3%) | | 0.788 | |
|  | No | 33(68.8%) | | 104(66.7%) | |  |  |
| Glycemic control | Good | 29(60.4%) | | 42(26.9%) | | P<0.001 | |
|  | Poor | 19(39.6%) | | 114(73.1%) | |  |  |
| HDL | Normal(>40 mg/dl) | 36(75.0%) | | 68(43.6%) | | P<0.001 | |
|  | Abnormal( <40mg/dl) | 12(25.0%) | | 88(56.4%) | |  |  |
| LDL | Normal( <=100mg/dl) | 40(83.3%) | | 73(46.8%) | | P<0.001 | |
|  | Abnormal(>100mg/dl) | 8(16.7%) | | 83(53.2%) | |  |  |
| TC | normal (<200mg/dl) | 35(72.9%) | | 49(31.4%) | | P<0.001 | |
|  | abnormal (>200mg/dl) | 13(27.1%) | | 107(68.6%) | |  |  |
| TG | normal (<150mg/dl) | 26(54.2%) | | 66(42.3%) | | P=0.149 | |
|  | abnormal (>150mg/dl | 22(45.8%) | | 90(57.7%) | |  |  |
