# Supplementary material for: Prevalence and determinants of erectile dysfunction among type 2 diabetes mellitus patients at selected government hospitals in gurage zone: A cross-sectional study
Source: PLoS One. 2025 Apr 21;20(4):e0318908. doi: 10.1371/journal.pone.0318908 (PMC12011261; doi:10.1371/journal.pone.0318908)
Supplement: S1 File — (DOCX) [file pone.0318908.s002.docx]

**S File 1. QUESTIONNAIRE**

**INSTRUCTIONS:** This questionnaire is designed to“Prevalence and Determinants of Erectile Dysfunction among Type 2 Diabetes Mellitus patients at Selected Government Hospitals in Gurage Zone

**Direction**: if you have any concerns and a problem, don’t hesitate to let me know and I’ll do my best to help’**.** Please circle the patients’ responses from the given options.

***ANNEX 3.1: QUESTIONER IN ENGLISH VERSION***

**Participant Identification**

Participant serial number ________________ Identification code_______________

Participant address______________________ Phone number______________

Participant signature _______

Data collector name _________________ Date ___________________________ Signature

| s.no | **Part I: Socio Demographic** | Response |  |
| --- | --- | --- | --- |
| 01 | AGE | ______________year |  |
| 02 | Marital status | Single  Married  Separate  Divorced  Widowed |  |
| 03 | Educational status | 1.No formal education  2. Primary  3. Secondary  4. Diploma  5. Degree and Above |  |
| 04 | Occupational Status | Farmer  Merchant  Gov’t employee  Housewife  Other(s) ___ | |
| 05 | Monthly income in Ethiopian Birr |  | |
| **Part 2: Behavioral factors** | |  | |
| 06 | Do you drink alcohol? (such as beer, wine, Katikala, Tej, arake ) | 1. Yes 2.No | |
| 07 | Do you smoke a cigarette? | 1.Yes 2.No | |
| 08 | Do you chew khat(kata adulis forsik) ? | 1. yes 2. No | |
| 09 | Do you have regular physical activity ? (including walking) | 1.Yes 2.No | |

**Part 3: The IIEF-5 CHECKLIST** First, you could say “Many men (ED affects particular patient, such as diabetes, hypertension, medication, recent heart attack, etc.) experience sexual problems, then ask ‘’Has this happened to you?”. These questions ask about the effects that your erection problems have had on your sex life over the last 6 months. Please try to answer the questions as honestly and as clearly as you are able. Your answers will help your doctor to choose the most effective treatment suited to your condition

In answering the questions, the following definitions apply:

Sexual Activity: Includes intercourse, caressing, foreplay & masturbation

Sexual Intercourse: Is defined as sexual penetration of your partner

Sexual Stimulation: Includes situations such as foreplay, erotic pictures, etc.

| **Part 3 : The IIEF-5 CHEKLIST** | | |
| --- | --- | --- |
| **s.no** | **OVER THE PAST 6 MONTHS CHECK ONE BOX ONLY** | |
| 10 | **HOW DO YOU RATE YOUR CONFIDENCE THAT YOU COULD GET AND KEEP AN ERECTION?** | |
|  | Very low | 1 point |
|  | Low | 2 points |
|  | Moderate | 3 points |
|  | High | 4 points |
|  | Very high | 5 points |
| 11 | **WHEN YOU HAD ERECTIONS WITH SEXUAL STIMULATION, HOW OFTEN WERE YOUR ERECTIONS HARD ENOUGH FOR PENETRATION (ENTERING YOUR PARTNER)?** | |
|  | Almost never or never | 1 point |
|  | A few times(much less than half the time) | 2 points |
|  | Sometimes(about half the time) | 3 points |
|  | most times(much more than half the time) | 4 points |
|  | Almost always or always | 5 points |
| 12 | **DURING SEXUAL INTERCOURSE, HOW OFTEN WERE YOU ABLE TO MAINTAIN YOUR ERECTION AFTER YOU HAD PENETRATED YOUR PARTNER?** | |
|  | Almost never or never | 1 point |
|  | A few times(much less than half the time) | 2 points |
|  | Sometimes(about half the time) | 3 points |
|  | Most times(much more than half the time) | 4 points |
|  | Almost always or always | 5 points |
| 13 | **DURING SEXUAL INTERCOURSE, HOW DIFFICULT WAS IT TO MAINTAIN YOUR ERECTION TO COMPLETION OF INTERCOURSE?** | |
|  | Extremely difficult | 1 point |
|  | Very difficult | 2 points |
|  | Difficult | 3 points |
|  | Slightly difficult | 4 points |
|  | Not difficult | 5 points |
| 14 | **WHEN YOU ATTEMPTED SEXUAL INTERCOURSE, HOW OFTEN WAS IT SATISFACTORY TO YOU?** | |
|  | Almost never or never | 1 points |
|  | A few times(much less than half the time) | 2 points |
|  | Sometimes(about half the time) | 3 points |
|  | Most times(much more than half the time) | 4 points |
|  | Almost always or always | 5 points |

|  | **Acceptance and treatment-seeking rate for ED** | |
| --- | --- | --- |
| 15 | Have you ever seek any medical help for sexual-related problems before? | |
|  | 1.No 2.Yes |  |
| 16 | If ‘yes’ did you treated the sexual problem? | |
|  | 1.No 2. Yes |  |
| 17 | If ‘yes’ what type of sexual treatment do you follow? |  |
|  | 1.drug 2. Psychotherapy 3. Both drug & psychotherapy |  |
| 18 | Do you think that drugs are effective in resolving sexual problems in diabetics? | |
|  | 1.No 2.Yes | |
| 19 | Do you think sexual counseling is effective in resolving sexual problems in diabetics? | |
|  | 1.No 2.Yes | |

| **PART 4:Physical Measurements** | | **value** |
| --- | --- | --- |
| 20 | Blood Pressure (mmHg) |  |
| 21 | Weight (kg) |  |
| 22 | Height (m) |  |
| 23 | BMI (Kg/m^2^) |  |

| **Part 5**: **Medical history** | | **Alternatives** |
| --- | --- | --- |
| 24 | How long you have been diagnosed with DM? | -------------months/years |
| 25 | **Do you have any of DM complication?** | 1.Yes 2.No |
| 26 | If yes, for question No. 22 which DM complication Do you have? | Hypertension  Renal problem  Nerve problem  Eye problem  Other(specify)------------------ |
| 27 | Do you take anti-hypertensive drug? | 1.No 2.Yes |
| 28 | Latest(last) two Fasting Blood Sugar(FBS) value(mg/dl) | 1. ------------------------------  2.------------------------------ |

| **Laboratory result** | | |
| --- | --- | --- |
| **PART 6: Biochemical Measurements** | | **value** |
| 29 | Current Fasting Blood Sugar (FBS) of the patients | --------------------mg/dl |
|  | Result of lipid profile | |
| 30 | HDL(mg/dl) ‎ |  |
| 31 | LDL(mg/dl) ‎ |  |
| 32 | TC(mg/dl) ‎ |  |
| 33 | TG(mg/dl) ‎ |  |

Data collector name _________________ Date _________Signature

***Thank you for your cooperation!!!***

***Data Collector Name__________________________***

***Name of the Hospital____________***

***Signature ___________________Date_____________________***

***ANNEX 3.2: QUESTIONER IN AMHARIC VERSION***

የተሳታፊ መለያ

የተሳታፊ መለያ ቁጥር ________________ መለያ ኮድ _______

የተሳታፊ አድራሻ ______________________ ስልክ ቁጥር______________

የተሳታፊ ፊርማ _______

የመረጃ ሰብሳቢው ስም _________________ ቀን ___________________________ ፊርማ

ለመረጃ ሰብሳቢዎች መመሪያ እባክዎን ከተሰጡት አማራጮች የታካሚዎችን ምላሽ ይክብቡ ፡፡

| **ተቁ** | **ክፍል አንድ፡ የቤተሰብና ማህበራዊ ገጽታ** | **መልስ** |
| --- | --- | --- |
| 01 | ዕድሜ (ዓመት) |  |
| 02 | የጋብቻ ሁኔታ | ያላገባ  የተጋባ  የተለያየ  ተፋታ  ሚስት የሞተበት |
| 03 | የትምህርት ሁኔታ | 1.መደበኛ ትምህረት ያለተማረ  2. የመጀመሪያ ደረጃ  3. ሁለተኛ ደረጃ  4. ኮሌጅ  5. ዲግሪና ከዚያበላይ |
| 04 | የሚተዳደሩበት የስራ አይነት | 1.ገበሬ  2.ነጋዴ  3.የመንግስት ሰራተኛ  4.የቤት አባወራ  5.(ሌሎች) ________ |
|  | **ክፍል 2፡የሕይወት ዘይቤ ልምዶች** |  |
| 05 | ወርሃዊ ገቢ በኢትዮ. ብር |  |
| 06 | አልኮል ይጠጣሉ? | 1. አዎ 2.አልጠጣም |
| 07 | ሲጋራ ያጨሳሉ ? | 1. አዎ 2.አላጨሰም |
| 08 | የአካል ብቃት እንቅስቃሴ ያደርጋሉ ? | 1. አዎ 2. አላደርግም |
| 09 | ጫት ይቅማሉ? | 1. አዎ 2. አልቅምም |

**ክፍል 3. የግብረ ስጋ ግንኙነት** **ተግባር አለም አቀፍ መረጃ ጠቋሚ -5 መጠይቅ።**

እነዚህ ጥያቄዎች በአለፉት ስድስት ወራት ውስጥ የግብረ ስጋ ግንኙነት ተግባር ሕይወትዎ ላይ ምን ውጤት እንዳስገኙ ይጠይቃሉ ፡፡ እባክዎን ለጥያቄዎችዎ በሐቀኝነት እና በግልፅ መልስ ለመስጠት ይሞክሩ ፡፡ መልሶችዎ ዶክተርዎ ለእርስዎ ሁኔታ በጣም የሚስማማዎትን በጣም ውጤታማ ህክምናን ለመምረጥ ይረዳቸዋል ፡፡ ጥያቄዎቹን ለመመለስ የሚከተሉትን ትርጓሜዎች ተግባራዊ ይሆናሉ፡

- የግብረ ስጋ ግንኙነት እንቅስቃሴ=የግብረ ሥጋ ግንኙነትን ፣ ቅልጥፍናን ፣ ቅድመ እይታን ያጠቃልላል

- የግብረ ስጋ ግንኙነት ግንኙነት=የባልደረባዎ ግብረስጋዊ ግንኙነት ተብሎ ይገለጻል

- የግብረ ስጋ ግንኙነት ማነቃቂያ =እንደ የግብረ ስጋ ግንኙነት ማማቂያ ጫወታ ፣ የግብረ ስጋ ግንኙነት ሥዕሎች ወዘተ ያሉ ሁኔታዎችን ያጠቃልላል።

| **ተቁ** | **በአለፉት ስድስት ወራት ውስጥ አንድ ሳጥን ላይ ብቻ( √ ) ምልክት ያድርጉ** | |
| --- | --- | --- |
| 10 | **ብልቶ መቆም እና ማቆየት እንደሚችሉ ያለዎትን እምነት እንዴት ይለካሉ?** | |
|  | በጣም ዝቅተኛ | 1 ነጥብ |
|  | ዝቅተኛ | 2 ነጥብ |
|  | መካከለኛ | 3 ነጥብ |
|  | ከፍተኛ | 4 ነጥብ |
|  | በጣም ከፍተኛ | 5 ነጥብ |
| 11 | **በግብረ ስጋ ግንኙነት መነቃቃት ሰኣት ብልቶ ቆሞ አና ጥንካሪ ኖሮት በግብረ ስጋ ግንኙነት ወቅት ለምን ያህል ግዚ ይቆያሉ** | |
|  | በጭራሽ አይቆይም/አይቆይም | 1 ነጥብ |
|  | ጥቂት ጊዜያት (ከግማሽ ጊዜ በታች) | 2 ነጥብ |
|  | አንዳንድ ጊዜ (ግማሽ ጊዜ ያህል) | 3 ነጥብ |
|  | ብዙ ጊዜ (ከግማሽ ጊዜ በላይ) | 4 ነጥብ |
|  | ሁልጊዜ | 5 ነጥብ |
| 12 | **ግንኙነት በሚፈጽሙበት ወቅት ብልቶ ኣጋሮ ወስጥ ከገባ በሁላ ለምን ያህል ግዚ ብልቶ ቆሞ/ሳይተኛ ይቆያል?** | |
|  | ምንም አይቆምም | 1 ነጥብ |
|  | ጥቂት ጊዜያት (ከግማሽ ጊዜ በታች) | 2 ነጥብ |
|  | አንዳንድ ጊዜ (ግማሽ ጊዜ ያህል) | 3 ነጥብ |
|  | ብዙ ጊዜ (ከግማሽ ጊዜ በላይ) | 4 ነጥብ |
|  | ሁልጊዜ | 5 ነጥብ |
| 13 | **የግብረ ሥጋ ግንኙነትን በሚፈጽሙበት ወቅት የግብረ ሥጋ ግንኙነትዎን እስከ ማጠናቀቁ ድረስ ብልቶ ቆሞ ማቆየት ምን ያህል አስቸጋሪ ነበር?** | |
|  | እጅግ በጣም አስቸጋሪ | 1 ነጥብ |
|  | በጣም አስቸጋሪ | 2 ነጥብ |
|  | አስቸጋሪ ፡፡ | 3 ነጥብ |
|  | ጥቂት አስቸጋሪ | 4 ነጥብ |
|  | አስቸጋሪ አይደለም ፡፡ | 5 ነጥብ |
| 14 | **የግብረስጋ ግንኙነት በሚፈጽሙበት ጊዜ ምን ያህል ጊዜ ለእርስዎ አጥጋቢ/አርኪ ነበር?** | |
|  | በጭራሽ አልረካሁም/አልረካሁም | 1 ነጥብ |
|  | ጥቂት ጊዜያት (ከግማሽ ጊዜ በታች) | 2 ነጥብ |
|  | አንዳንድ ጊዜ (ግማሽ ጊዜ ያህል) | 3 ነጥብ |
|  | ብዙ ጊዜ (ከግማሽ ጊዜ በላይ) | 4 ነጥብ |
|  | ሁልጊዜ | 5 ነጥብ |

|  |  | |
| --- | --- | --- |
|  | **ክፍል 4: - ለብልት አለመቆም የሚደረግ ህክምና የመቀበል እና የመፈለግ ሁኒታ መጠይቆች** | |
| 15 | ከዚህ በፊት ከ ግብረ ስጋ ግንኙነት ጋር ለተያያዙ ችግሮች ማንኛውንም የሕክምና ዕርዳታ ጠይቀዋል? | |
|  | 1. አዎ 2. አልጠየኩም |  |
| 16 | 'አዎ' ብለው ከሆነ ለ ግብረስጋዊ ችግሩ ህክምና አደረጉ? | |
|  | 1. አዎ 2. አላደረኩም |  |
| 17 | አዎ' ብለው ከሆነ የትኛው አይነት ለ ግብረስጋዊ ችግሩ ህክምና አደረጉ? |  |
|  | 1.የመድሃኒት 2.የ ስነ-ልቦና 3. ሁለቱም የመድሃኒት እና የስነ-ልቦና ህክምና |  |
| 18 | በስኳር ህመምተኞች ውስጥ የሩካቢ ችግሮችን ለመፍታት መድኃኒቶች ውጤታማ ናቸው ብለው ያስባሉ? | |
|  | . 1. አዎ 2. አላስብም | |
| 19 | ስለ ጾታዊ ግንኙነት ማማከር በስኳር ህመምተኞች ውስጥየሩካቢ ችግሮችን ለመፍታት ውጤታማ ነው ብለው ያስባሉ? | |
|  | 1. አዎ 2. አላስብም | |

|  | | |  |  |
| --- | --- | --- | --- | --- |
|  | **ክፍል 5 ፡የሰዉነት ልኬት** | | መጠን | |
| 20 | **የደም ግፊት መጠን (mmHg)** | |  | |
| 21 | ቁመት (ሜትር) | |  | |
| 22 | ክብደት ( ኪ.ግራም) | |  | |
| 23 | **የሰዉነት አቋም መጠን(ኪ.ግ /ሜ2)** | |  | |
| ክፍል 3፡የጢና ሁኒታ | | | አማራጭ |  |
| 24 | | የስኳር ሕመም እንዳለቦት ካወቁ ምን ያህል ግዜ ሆኖታል? |  |  |
| 25 | | ከስኳር የሕመም ጋር ተያያዥነት ያላቸው በሽታዎች አለቦዎት? | 1. አዎ 2 .የለብኝም |  |
| 26 | | አዎ ከሆነ መልሶ; የትኛው አይነት ከስኳር ሕመም ጋር ተያያዥነት ያለው በሽታ አለቦት? | 1. የደም ግፊት  2.የኩላሊት ችግር  3.የአእምሮ ህመም ችግር  4.የአይን እይታ ችግር  5.ሌላ ካላ ግለጽ ------------------ |  |
| 27 | | ለደም ግፊት ህመም መድሃኒት ይወስዳሉ? | 1. አዎ 2 . አልወስድም |  |
| 28 | | የቅርብ ግዜ የሁለት ተከታታይ የስኳር መጠን ልኬት |  |  |

| **PART 6: የላቦራቶሪ ዉጤት** | | **value** |
| --- | --- | --- |
| 29 | Current Fasting Blood Sugar (FBS) of the patients | --------------------mg/dl |
|  | Result of lipid profile | |
| 30 | HDL(mg/dl) ‎ |  |
| 31 | LDL(mg/dl) ‎ |  |
| 32 | TC(mg/dl) ‎ |  |
| 33 | TG(mg/dl) ‎ |  |

*ለትብብርዎ እናመሰግናለን!!!

የመረጃ ሰብሳቢው ስም ___________________________

የሆስፒታሉ ስም _______

ፊርማ___________________ ቀን______________________
