# Supplementary material for: Prevalence and determinants of erectile dysfunction among type 2 diabetes mellitus patients at selected government hospitals in gurage zone: A cross-sectional study
Source: PLoS One. 2025 Apr 21;20(4):e0318908. doi: 10.1371/journal.pone.0318908 (PMC12011261; doi:10.1371/journal.pone.0318908)
Supplement: Attachment — Submitted filename: Response to Reviewers.docx [file pone.0318908.s005.docx]

To,

The Editor, Reviewers,

PLOS ONE

Date: September 9, 2024

# Subject: Addressing Editor/Reviewer Comments for PONE-D-24-27116

Dear Editor, Reviewers,

Many thanks to you for your helpful feedback.We have now tried to address all your concerns to the best of our abilities. Kindly help us with your further concerns (if any) to improvise our paper. Below we are providing our responses to the comments and actions that we took to address them. The responses are highlighted in blue.

Date:	Sep 10, 2024

Thank you. Kind regards,

Authors of PONE-D-24-27116

PONE-D-24-27116

Prevalence and Determinants of Erectile Dysfunction among Type 2 Diabetes Mellitus patients at Selected Government Hospitals in Gurage Zone

PLOS ONE

Dear Dr. ABRAR,

Thank you for submitting your manuscript to PLOS ONE. After careful consideration, we feel that it has merit but does not fully meet PLOS ONE’s publication criteria as it currently stands. Therefore, we invite you to submit a revised version of the manuscript that addresses the points raised during the review process.

==============================

ACADEMIC EDITOR: Thanks for submitting this manuscript to this esteemed journal. Based on the 3 reviews especially addressing the novelty about what this research adds to what is already known i suggest drafting and major revision Thanks ;

==============================

Please submit your revised manuscript by Oct 16 2024 11:59PM. If you will need more time than this to complete your revisions, please reply to this message or contact the journal office at plosone@plos.org. When you're ready to submit your revision, log on to https://www.editorialmanager.com/pone/ and select the 'Submissions Needing Revision' folder to locate your manuscript file.

Please include the following items when submitting your revised manuscript:

- A rebuttal letter that responds to each point raised by the academic editor and reviewer(s). You should upload this letter as a separate file labeled 'Response to Reviewers'.
- Included
- A marked-up copy of your manuscript that highlights changes made to the original version. You should upload this as a separate file labeled 'Revised Manuscript with Track Changes'.
- Included
- An unmarked version of your revised paper without tracked changes. You should upload this as a separate file labeled 'Manuscript'.
- Included

If you would like to make changes to your financial disclosure, please include your updated statement in your cover letter. Guidelines for resubmitting your figure files are available below the reviewer comments at the end of this letter.

If applicable, we recommend that you deposit your laboratory protocols in protocols.io to enhance the reproducibility of your results. Protocols.io assigns your protocol its own identifier (DOI) so that it can be cited independently in the future. For instructions see: https://journals.plos.org/plosone/s/submission-guidelines#loc-laboratory-protocols. Additionally, PLOS ONE offers an option for publishing peer-reviewed Lab Protocol articles, which describe protocols hosted on protocols.io. Read more information on sharing protocols at https://plos.org/protocols?utm_medium=editorial-email&utm_source=authorletters&utm_campaign=protocols.

We look forward to receiving your revised manuscript.

Kind regards,

Kamal Sharma

Academic Editor

PLOS ONE

Journal Requirements:

When submitting your revision, we need you to address these additional requirements.

1. Please ensure that your manuscript meets PLOS ONE's style requirements, including those for file naming. The PLOS ONE style templates can be found at

https://journals.plos.org/plosone/s/file?id=wjVg/PLOSOne_formatting_sample_main_body.pdf and

https://journals.plos.org/plosone/s/file?id=ba62/PLOSOne_formatting_sample_title_authors_affiliations.pdf

2. We note that the grant information you provided in the ‘Funding Information’ and ‘Financial Disclosure’ sections do not match.

When you resubmit, please ensure that you provide the correct grant numbers for the awards you received for your study in the ‘Funding Information’ section.

We matched the grant information you provided in the ‘Funding Information’ and ‘Financial Disclosure’ sections.

3. Thank you for stating the following financial disclosure:

"This research work was supported by Jimma university school of medical laboratory sciences,

faculty of health science, and institute of health. The research received no funding and no

specified grant number."

Please state what role the funders took in the study. If the funders had no role, please state: ""The funders had no role in study design, data collection and analysis, decision to publish, or preparation of the manuscript.""

If this statement is not correct you must amend it as needed.

Please include this amended Role of Funder statement in your cover letter; we will change the online submission form on your behalf.

The statement is amended and The research received no funding and no specified grant number.

4. Your ethics statement should only appear in the Methods section of your manuscript. If your ethics statement is written in any section besides the Methods, please move it to the Methods section and delete it from any other section. Please ensure that your ethics statement is included in your manuscript, as the ethics statement entered into the online submission form will not be published alongside your manuscript.

Ethical statement is moved to the methods section and delete it from any other section.Kindly

include the following text on the online submission form

5. Please ensure that you refer to Figure 1 in your text as, if accepted, production will need this reference to link the reader to the figure.

The figure 1 is cited in the text reference to the link

6. Please include a copy of Table 3 and 5 which you refer to in your text on page 13 and 16.

The copy of table 3 and 5 is cited in the text reference to the link belongs to the page

7. Please include captions for your Supporting Information files at the end of your manuscript, and update any in-text citations to match accordingly. Please see our Supporting Information guidelines for more information: http://journals.plos.org/plosone/s/supporting-information.

Supporting Information files were captioned at the end of the manuscript, and updated any in-text citations to match according to Supporting Information guidelines : http://journals.plos.org/plosone/s/supporting-information.

Additional Editor Comments:

Hello,

Based on the reviewers comments the manuscript needs to undergo a major revision addressing all the issues raised by all the 3 reviewers

Thanks

[Note: HTML markup is below. Please do not edit.]

Reviewers' comments:

Reviewer's Responses to Questions

Comments to the Author

1. Is the manuscript technically sound, and do the data support the conclusions?

The manuscript must describe a technically sound piece of scientific research with data that supports the conclusions. Experiments must have been conducted rigorously, with appropriate controls, replication, and sample sizes. The conclusions must be drawn appropriately based on the data presented.

Reviewer #1: Yes

Reviewer #2: Yes

Reviewer #3: Yes

2. Has the statistical analysis been performed appropriately and rigorously?

Reviewer #1: Yes

Reviewer #2: No

Reviewer #3: Yes

3. Have the authors made all data underlying the findings in their manuscript fully available?

The PLOS Data policy requires authors to make all data underlying the findings described in their manuscript fully available without restriction, with rare exception (please refer to the Data Availability Statement in the manuscript PDF file). The data should be provided as part of the manuscript or its supporting information, or deposited to a public repository. For example, in addition to summary statistics, the data points behind means, medians and variance measures should be available. If there are restrictions on publicly sharing data—e.g. participant privacy or use of data from a third party—those must be specified.

Reviewer #1: Yes

Reviewer #2: Yes

Reviewer #3: Yes

4. Is the manuscript presented in an intelligible fashion and written in standard English?

PLOS ONE does not copyedit accepted manuscripts, so the language in submitted articles must be clear, correct, and unambiguous. Any typographical or grammatical errors should be corrected at revision, so please note any specific errors here.

Reviewer #1: Yes

Reviewer #2: Yes

Reviewer #3: Yes

5. Review Comments to the Author

Please use the space provided to explain your answers to the questions above. You may also include additional comments for the author, including concerns about dual publication, research ethics, or publication ethics. (Please upload your review as an attachment if it exceeds 20,000 characters)

Reviewer #1: Please, in the abstract, method, instead of 204 diabetic patients, the wording should be corrected as 204 diabetic men. In the results, the same should be corrected.

**We have now revised the content as per suggestion.**

Two age groups under and over 40 years were considered. Please divide the age group above 40 years into subgroups and compare the prevalence between them

**We have now revised the content as per suggestion, the age grouped .< 40 years ,40-50 years and >50 years and the prevalence were computed**

In the results, instead of blood sugar above 7, write A1C. Because this is how the method is perceived.

Limitations should be mentioned.

**We have now revised the content as per suggestion and the Limitations also were mentioned.**

Reviewer #2: General comments

This is an interesting study which highlighted the prevalence and predictors of ED among male patients with diabetes in three hospital in Ethiopia. However, a few issues may need to be addressed to strengthen the paper.

Specific comments

1. Page 6, sample size determination: How representative is a convenience sample of 204 male type 2 DM patients in the study areas? It will be important to state the estimated number of male type 2 DM patients that usually attend the AH, BH and WKUSH hospitals in a four months period. Please include the sample size formula and the variables used for the calculation of sample size, so the reader can follow your calculation.

**We have now revised the content as per suggestion. estimated number of male type 2 DM patients Over the 4-month study period, 104 type 2 diabetic patients were recruited from BH, 60 from WKUSH, and 40 from AH. the sample size was computed as:**

**n= (Z) ^2^ p (1-p) /d^2^	; n= [(1.96) ²* 0.8439*(1-0.8439)]/(0.05)^2^ = 204**

2.Page 7, exclusion criteria, figure 1:

a.1st line: The statement “Study participants with the following disorder will exclude from the study” should read “Study participants with the following disorders were excluded from the study”

Thank you, we have now corrected

b.“Exclusion criterias” should read “Exclusion criteria” since criteria is already a plural word.

Thank you, we have now corrected

c.Menatlly Incompitent: has spelling error. Should read “Mentally incompetent”. However, what do you mean by mentally incompetent? Please rephrase for clarity.

Thank you, we have now corrected and repharesed to “Cognitively impaired”

d.History of Deperession: should read “History of Depression”.

Thank you, we have now corrected

e.Overt Psychlogical Disorder: should read “Overt Psychological Disorder”

Thank you, we have now corrected

f.“Nurological” should be replaced by “Neurological”

Thank you, we have now corrected **.**

2. Page 9. Data collection, line 3: what type of consent did you obtain from the participants? Was it written or verbal? This should be clearly stated.

**We have now revised the content as per suggestion oral consent were obtained from the participants and clearly stated**

3. Page 9, 4.7.1.1, socio-demographic, anthropometric and related clinical data: It will be important to clearly define smoking status, alcohol consumption, exercise status, and income. What is currently stated is still unclear. For instance, when you say smoker and non-smoker, what do you mean? Do you mean never ever smoked, stopped smoking 6 months ago, actively smoking, etc. There are standard definitions for these words and needs to be stated as such and also cite appropriate references from the literature to back your definitions. This will help the reproducibility of the study.

**Thank you, we have now included standard definitions of the words and stated as such and cited with appropriate references**

RESULTS

4. Table 1, educational and occupational statuses, frequency columns do not total 100%. Please cross check.

**Thank you, We have now cross checked and corrected**

6.Page 13, 5.3 prevalence of erectile dysfunction among the study participants:

a.line 1: If 204 male T2DM patients were interviewed, how come 216 had T2DM and ED? Do you mean 156 instead of 216? Please cross-check.

**Thank you, We have now revised and corrected to 156**

b.Lines 3-6: “Further analysis of the data showed that 48 men had no ED, 15 had mild ED, 28 had mild-to-moderate ED, 46 had moderate ED, and 67 had severe ED. The data also indicated that a maximum of 32.8% of men with diabetes had severe ED, while only 7.4% had a mild form of ED..” It will be important to include both the numbers and the full percentages of the ED severity types because it appears the figure presented shows summarized percentages.

**Thank you, We have now revised and included both the numbers and the full percentages of the ED severity**

**The prevalence rate of ED among Type 2 diabetic men was 76.5% (156 out of 204). Further analysis of the data showed that 48(23%) men had no ED, 15(7%) had mild ED, 28(14%) had mild-to-moderate ED, 46(23%) had moderate ED, and 67(33%) had severe ED**

7. Page 14, Table 4: what test was employed to determine the association between clinical variables and ED among study participants? From this table, it appears you applied a different statistical test other than bivariate logistic regression. It seems you used a chi-square test. Please clarify and possibly re-write your section 5.4.

**Thank you, We have now revised and corrected**

**Chi-square test was carried out to discover the relationship between two categorical variables and re-wrote in the section**

DISCUSSION

8. Page 19, paragraph 3: No need to restate the AOR, 95%CI and P value in the discussion. This also applies to the last paragraph and elsewhere.

**Thank you dear reviewer for your helpful comments, We have now revised and restated the content as per suggestion.**

9.Strength and limitations of the study: the convenience sampling technique presents a limitation towards generalising the study result. Given the selection bias, those who participated may be significantly different from those who did not.

**Thank you, dear reviewer. We have now revised and restated the content as per suggestion.**

Reviewer #3: This is a work about the prevalence and determinants of ED among T2D patients at Selected Government Hospitals in Gurage Zone. Anyway, no novelties about this topic are producted. I have comments and suggestions.

Abstract

- please, as suggested by international guidelines, replace here and in the manuscript, diabetic with subjects/patients with diabetes (T2D).

**Thank you, We have now revised and replaced the abstract according to the guideline in the manuscript**

**ABSTRACT**

**Background**: - Erectile dysfunction (ED) is often not recognized in men with type 2 diabetes mellitus (T2DM), despite the prevalence of the disease. Early diagnosis of ED in T2DM is very important for effective treatment and prevention of serious complications such as cardiovascular events. This study investigates the Prevalence and Determinants of Erectile Dysfunction among Type 2 Diabetes Mellitus patients at Selected Government Hospitals in Gurage Zone.

**Methods:** - A study was conducted in three public hospitals from September 1 to December 30, 2020. 204 diabetic men were selected using consecutive sampling. Erectile function was evaluated utilizing the International Index of Erectile Function-5. HbA1c and lipid levels were analyzed utilizing a Cobas 600 clinical chemistry analyzer. The data was analyzed using the Statistical Package for Social Sciences-20.

**Results:** -ED was found in 156 (76.5%) diabetic males. Age above 40 years, alcohol consumption, khat chewing, overweight, obesity, duration of diabetes greater than 5 years , uncontrolled diabetes, high low-density lipoprotein , and high total cholesterol (P < 0.05) were independent predictors of ED.

**Conclusion :** The study has confirmed that there is a high prevalence of erectile dysfunction among male with type 2 diabetes in the study settings. As a result, clinicians need to take preventive measures and work on unveiling the factors that cause ED in these patients.

**Keywords: Type 2 Diabetes Mellitus, Erectile Dysfunction, Gurage**

Introduction

- "it remains uncertain whether erectile dysfunction (ED) in diabetic males stems

solely from hyperglycemia and microvascular complications or arises from a combination of

factors such as cardiovascular diseases, hypertension, smoking, and obesity". Vs 2010, now we have more scientific data to prove these combinations.

Thank you, dear reviewer. **We have now included the content as per suggestion**

Materials and methods ( very long! )

Thank you for pointing this out. We have tried to minimize the emphasized

portions.

Inclusion/exclusion criteria: You insert T2D as an exclusioon criteria. Please, check!

**Thank you, We have now removed from exclusion criteria**

Discussion

- some parts of results are here too.

**Thank you, We have now removed parts of results**

- please, clarify better strenghts and limitations of this work, not after conclusions section.

**Thank you dear reviewer for these helpful pointers. We have now better clarified strengths and limitations of this work and inserted before conclusions section.**

Editorial guidelines needs to be checked.

Check acronyms as type 2 diabetes

6. PLOS authors have the option to publish the peer review history of their article (what does this mean?). If published, this will include your full peer review and any attached files.

If you choose “no”, your identity will remain anonymous but your review may still be made public.

Do you want your identity to be public for this peer review? For information about this choice, including consent withdrawal, please see our Privacy Policy.

Reviewer #1: Yes: Maryam Zahedi, Assistant Professor of Endocrinology & Metabolism,

Clinical Research Development Unit (CRDU),

Sayad Shirazi Hospital,

Golestan University of Medical Sciences,

Gorgan, Iran.

Tel: +98 9111778058

Email: drmaryam.zahedi@yahoo.com

Reviewer #2: Yes: Godpower Chinedu Michael

Reviewer #3: No

[NOTE: If reviewer comments were submitted as an attachment file, they will be attached to this email and accessible via the submission site. Please log into your account, locate the manuscript record, and check for the action link "View Attachments". If this link does not appear, there are no attachment files.]

While revising your submission, please upload your figure files to the Preflight Analysis and Conversion Engine (PACE) digital diagnostic tool, https://pacev2.apexcovantage.com/. PACE helps ensure that figures meet PLOS requirements. To use PACE, you must first register as a user. Registration is free. Then, login and navigate to the UPLOAD tab, where you will find detailed instructions on how to use the tool. If you encounter any issues or have any questions when using PACE, please email PLOS at figures@plos.org. Please note that Supporting Information files do not need this step.

In compliance with data protection regulations, you may request that we remove your personal registration details at any time. (Remove my information/details). Please contact the publication office if you have any questions.
