# Supplementary material for: Prevalence and determinants of erectile dysfunction among type 2 diabetes mellitus patients at selected government hospitals in gurage zone: A cross-sectional study
Source: PLoS One. 2025 Apr 21;20(4):e0318908. doi: 10.1371/journal.pone.0318908 (PMC12011261; doi:10.1371/journal.pone.0318908)
Supplement: Attachment — Submitted filename: Plos one review.docx [file pone.0318908.s004.docx]

with respect

I am very grateful to be allowed to be a reviewer of the valuable Plos One journal.

I enjoyed reading this article. A well-chosen topic that is useful.

Here are some things to improve the article, which I hope will be corrected before acceptance:

- Is it necessary to include the word garage in the keyword section?

- In the introduction, at the end of the second paragraph, instead of 1.3 million diabetics, 1.3 million diabetics should be added.

- In the text of the article, it is better to mention diabetes in one form, for example, diabetes mellitus, or preferably after the first time as DM.

- In the summary of the results, instead of saying that these factors were known as factors leading to erectile dysfunction, according to the nature of the article, only more accompaniment should be mentioned.

- In the exit criteria, "mentally incompetent" has spelling forms that should be corrected.

- Why is type 2 diabetes written in the exit criteria? This is an apparent mistake that needs to be corrected because you have precisely done the study on people with type 2 diabetes. Maybe you mean type 1 diabetes?

- As it was said about the results in the summary of the article, in the discussion about the factors associated with erectile dysfunction, do not use terms that mean causality and only mention the association, for example, about age.

- Please write down which edition of ED you used to define diabetes control. Although the definition may not have changed clearly, you must tell me which version you used.

- In the analyses, if available, it would have been better to use the continuous values of the variables to check their relationship with erectile dysfunction.
