# Supplementary material for: Prevalence and determinants of erectile dysfunction among type 2 diabetes mellitus patients at selected government hospitals in gurage zone: A cross-sectional study
Source: PLoS One. 2025 Apr 21;20(4):e0318908. doi: 10.1371/journal.pone.0318908 (PMC12011261; doi:10.1371/journal.pone.0318908)
Supplement: Attachment — Submitted filename: R2-response to reviwer_051814.docx [file pone.0318908.s006.docx]

To,

The Editor, Reviewers,

PLOS ONE

Date: October 23, 2024

**Subject: Addressing Editor/Reviewer Comments for PONE-D-24-27116R1**

Dear Editor, Reviewers,

Many thanks to you for your helpful feedback.We have now tried to address all your concerns to the best of our abilities. Kindly help us with your further concerns (if any) to improvise our paper. Below we are providing our responses to the comments and actions that we took to address them. The responses are highlighted in blue.

Date:	October 23, 2024

Thank you. Kind regards,

Authors of PONE-D-24-27116R1

PONE-D-24-27116R1

Prevalence and Determinants of Erectile Dysfunction among Type 2 Diabetes Mellitus patients at Selected Government Hospitals in Gurage Zone

PLOS ONE

Dear Dr. ABDLSHIKURE ,

Thank you for submitting your manuscript to PLOS ONE. After careful consideration, we feel that it has merit but does not fully meet PLOS ONE’s publication criteria as it currently stands. Therefore, we invite you to submit a revised version of the manuscript that addresses the points raised during the review process.

==============================

Hello,

The authors need to address the important points raised by our reviewers even though they have revised many of the previous aspects.

1. In the results section, the prevalence of ED in a previous study used for their calculation of sample size as 84.39%; they affirm that it is important to include the decimal places. However, they fail to provide such information here. The information in both Figure 2 and the text is incomplete or rather incorrect and misleading, which requires correction. For instance, the “no ED as 48 (23%)” should be “no ED as 48 (23.5%)”. The authors cannot trivialise this matter, given the status of this journal.

2. The authors did not appear to recognise that the discussion section is to interpret the study results (what do findings mean in the context of current evidence, practice and policy?) and not to state ODDS RATIO or sections of the result here.

Please address the same before acceptance. Thanks

==============================

Please submit your revised manuscript by Dec 02 2024 11:59PM. If you will need more time than this to complete your revisions, please reply to this message or contact the journal office at plosone@plos.org. When you're ready to submit your revision, log on to https://www.editorialmanager.com/pone/ and select the 'Submissions Needing Revision' folder to locate your manuscript file.

Please include the following items when submitting your revised manuscript:

- A rebuttal letter that responds to each point raised by the academic editor and reviewer(s). You should upload this letter as a separate file labeled 'Response to Reviewers'.
- Included
- A marked-up copy of your manuscript that highlights changes made to the original version. You should upload this as a separate file labeled 'Revised Manuscript with Track Changes'.
- Included
- An unmarked version of your revised paper without tracked changes. You should upload this as a separate file labeled 'Manuscript'.
- Included

If you would like to make changes to your financial disclosure, please include your updated statement in your cover letter. Guidelines for resubmitting your figure files are available below the reviewer comments at the end of this letter.

If applicable, we recommend that you deposit your laboratory protocols in protocols.io to enhance the reproducibility of your results. Protocols.io assigns your protocol its own identifier (DOI) so that it can be cited independently in the future. For instructions see: https://journals.plos.org/plosone/s/submission-guidelines#loc-laboratory-protocols. Additionally, PLOS ONE offers an option for publishing peer-reviewed Lab Protocol articles, which describe protocols hosted on protocols.io. Read more information on sharing protocols at https://plos.org/protocols?utm_medium=editorial-email&utm_source=authorletters&utm_campaign=protocols.

We look forward to receiving your revised manuscript.

Kind regards,

Kamal Sharma

Academic Editor

PLOS ONE

Journal Requirements:

Please review your reference list to ensure that it is complete and correct. If you have cited papers that have been retracted, please include the rationale for doing so in the manuscript text, or remove these references and replace them with relevant current references. Any changes to the reference list should be mentioned in the rebuttal letter that accompanies your revised manuscript. If you need to cite a retracted article, indicate the article’s retracted status in the References list and also include a citation and full reference for the retraction notice.

Thank you for the reminder regarding the reference list. We have thoroughly reviewed all the references to ensure their completeness and accuracy. We revised the reference based on the following considerations:

1. Relevance and Timeliness: The new reference provides more recent data and insights that align better with the scope of our research, ensuring that our work reflects the most up-to-date findings.

2. Improved Contextual Fit: Upon re-evaluating the original reference, we found that the newly cited work offers a clearer and more relevant theoretical or empirical basis for our arguments. This enhances the clarity and rigor of our manuscript.

3. Methodological or Regional Relevance: The new reference reflects a methodology, context, or geographic focus that aligns more closely with the objectives of our study.

We are confident that this change strengthens the manuscript and improves its overall coherence. Thank you again for your thorough review and constructive suggestions.

If the journal identifies any further issues with the references, we are happy to address them promptly.These updates are reflected in the revised manuscript and reference list.

Additional Editor Comments (if provided):

Hello,

The authors need to address the important points raised by our reviewers even though they have revised many of the previous aspects.

1. In the results section, the prevalence of ED in a previous study used for their calculation of sample size as 84.39%; they affirm that it is important to include the decimal places. However, they fail to provide such information here. The information in both Figure 2 and the text is incomplete or rather incorrect and misleading, which requires correction. For instance, the “no ED as 48 (23%)” should be “no ED as 48 (23.5%)”. The authors cannot trivialise this matter, given the status of this journal.

2. The authors did not appear to recognise that the discussion section is to interpret the study results (what do findings mean in the context of current evidence, practice and policy?) and not to state ODDS RATIO or sections of the result here.

Please address the same before acceptance. Thanks

[Note: HTML markup is below. Please do not edit.]

Reviewers' comments:

Reviewer's Responses to Questions

Comments to the Author

1. If the authors have adequately addressed your comments raised in a previous round of review and you feel that this manuscript is now acceptable for publication, you may indicate that here to bypass the “Comments to the Author” section, enter your conflict of interest statement in the “Confidential to Editor” section, and submit your "Accept" recommendation.

Reviewer #1: (No Response)

Reviewer #2: (No Response)

Reviewer #3: All comments have been addressed

2. Is the manuscript technically sound, and do the data support the conclusions?

The manuscript must describe a technically sound piece of scientific research with data that supports the conclusions. Experiments must have been conducted rigorously, with appropriate controls, replication, and sample sizes. The conclusions must be drawn appropriately based on the data presented.

Reviewer #1: (No Response)

Reviewer #2: Partly

Reviewer #3: Yes

3. Has the statistical analysis been performed appropriately and rigorously?

Reviewer #1: (No Response)

Reviewer #2: Yes

Reviewer #3: Yes

4. Have the authors made all data underlying the findings in their manuscript fully available?

The PLOS Data policy requires authors to make all data underlying the findings described in their manuscript fully available without restriction, with rare exception (please refer to the Data Availability Statement in the manuscript PDF file). The data should be provided as part of the manuscript or its supporting information, or deposited to a public repository. For example, in addition to summary statistics, the data points behind means, medians and variance measures should be available. If there are restrictions on publicly sharing data—e.g. participant privacy or use of data from a third party—those must be specified.

Reviewer #1: (No Response)

Reviewer #2: No

Reviewer #3: Yes

5. Is the manuscript presented in an intelligible fashion and written in standard English?

PLOS ONE does not copyedit accepted manuscripts, so the language in submitted articles must be clear, correct, and unambiguous. Any typographical or grammatical errors should be corrected at revision, so please note any specific errors here.

Reviewer #1: (No Response)

Reviewer #2: Yes

Reviewer #3: Yes

6. Review Comments to the Author

Please use the space provided to explain your answers to the questions above. You may also include additional comments for the author, including concerns about dual publication, research ethics, or publication ethics. (Please upload your review as an attachment if it exceeds 20,000 characters)

Reviewer #1: with respect

I am very grateful to be allowed to be a reviewer of the valuable Plos One journal.

I enjoyed reading this article. A well-chosen topic that is useful.

Here are some things to improve the article, which I hope will be corrected before acceptance:

- Is it necessary to include the word Gurage in the keyword section?

Thank you for your observation. We have revised the relevance of including the term "Gurage" in the keywords. we removed term "Gurage" and we added the word “Determinants”in the keywords.

In the introduction, at the end of the second paragraph, 1.3 million diabetics should be added instead of 1.3 million diabetes.

Thank you for pointing out this mistake. We have corrected the phrase to "1.3 million diabetics" to ensure accuracy.

- In the text of the article, it is better to mention diabetes in one form, for example, diabetes mellitus, or preferably after the first time as DM.

Thank you for the valuable suggestion. We have revised the manuscript to ensure consistency by using the term "diabetes mellitus" at first mention, followed by the abbreviation "DM" throughout the remainder of the text.

- According to the article's cross-sectional nature, the abstract's results should mention only association instead of saying that these factors were known as predisposing factors leading to erectile dysfunction.

Thank you for the insightful comment. We have revised the abstract to ensure that the results emphasize associations rather than implying causality, reflecting the cross-sectional nature of the study.

- In the exclusion criteria, "mentally incompetent" has spelling forms that should be corrected.

We appreciate your careful review. The term "mentally incompetent" has been corrected and paraphrased to “Cognitively impaired”

- Why is type 2 diabetes written in the exclusion criteria? This is an apparent mistake that needs to be corrected because you have precisely done the study on people with type 2 diabetes. Maybe you mean type 1 diabetes?

Thank you for catching this oversight. You are correct—our intention was to exclude individuals with type 1 diabetes. We have corrected the exclusion criteria accordingly.

- As it was said about the results in the article's abstract, in the discussion about the factors associated with erectile dysfunction, do not use terms that mean causality and only mention the association, for example, about age.

We appreciate your observation. In line with your suggestion, we have revised the discussion section to avoid causal language and ensure that the associations are clearly stated, particularly regarding the relationship between dependent and independent variables.

- Please write down which edition of ADA you used to define diabetes control. Although the definition may not have changed clearly, you should clarify which version you used.

Thank you for the suggestion. We have now specified the edition of the American Diabetes Association (ADA) guidelines used to define diabetes control in the methods section for clarity.We used “Association GoAD. Standards of medical care in diabetes–2015: summary of revisions. Diabetes Care. 2015;38:S4.”

- In the analyses, if available, it would have been better to use the continuous values of the variables to check their relationship with erectile dysfunction.

We appreciate this recommendation. Where appropriate, we have considered using continuous variables in the analysis. However, due to the data was collected in group or categorical form,continues values were not recorded ,making them inaccessible for continuous data analysis, we opted to categorize certain variables for this study. We will take this valuable input into account in future studies to enhance the robustness of our analysis.

Reviewer #2: 1. In the results section, the authors have not corrected the summarised results I mentioned. For instance, the authors cited the prevalence of ED in a previous study used for their calculation of sample size as 84.39%; they affirm that it is important to include the decimal places. However, they fail to provide such information here. The information in both Figure 2 and the text is incomplete or rather incorrect and misleading, which requires correction. For instance, the “no ED as 48 (23%)” should be “no ED as 48 (23.5%)”. The authors cannot trivialise this matter, given the status of this journal.

Thank you for your detailed review and for pointing out these discrepancies. We have revised the results section to ensure consistency and have included decimal places where appropriate, including in the prevalence values. Additionally, we have corrected the information in both Figure 2 and the corresponding text to read "no ED as 48 (23.5%)" to accurately reflect the data.

2. The authors did not appear to recognise that the discussion section is to interpret the study results (what do findings mean in the context of current evidence, practice and policy?) and not to state ODDS RATIO or sections of the result here. This issue was not resolved in their response and should also not be trivialised.

We appreciate your reminder about the purpose of the discussion section. We have now revised this section to focus on the interpretation of our findings in light of existing evidence, practice, and policy. References to odds ratios and other detailed results have been moved to the results section to ensure that the discussion aligns with the journal’s expectations.

Reviewer #3: All the queries were fully replied. Please, revise better the English language before to complete the sumbission.

Thank you for your positive feedback. We have carefully reviewed the manuscript to improve the clarity and correctness of the English language, ensuring it meets the journal’s standards.

7. PLOS authors have the option to publish the peer review history of their article (what does this mean?). If published, this will include your full peer review and any attached files.

If you choose “no”, your identity will remain anonymous but your review may still be made public.

Do you want your identity to be public for this peer review? For information about this choice, including consent withdrawal, please see our Privacy Policy.

Reviewer #1: No

Reviewer #2: Yes: Godpower Chinedu Michael

Reviewer #3: No

[NOTE: If reviewer comments were submitted as an attachment file, they will be attached to this email and accessible via the submission site. Please log into your account, locate the manuscript record, and check for the action link "View Attachments". If this link does not appear, there are no attachment files.]

While revising your submission, please upload your figure files to the Preflight Analysis and Conversion Engine (PACE) digital diagnostic tool, https://pacev2.apexcovantage.com/. PACE helps ensure that figures meet PLOS requirements. To use PACE, you must first register as a user. Registration is free. Then, login and navigate to the UPLOAD tab, where you will find detailed instructions on how to use the tool. If you encounter any issues or have any questions when using PACE, please email PLOS at figures@plos.org. Please note that Supporting Information files do not need this step.

In compliance with data protection regulations, you may request that we remove your personal registration details at any time. (Remove my information/details). Please contact the publication office if you have any questions.
